# AIRE Deficiency Impairs Thymic Epithelial Progenitor Differentiation from Mouse Embryonic Stem Cells and Accelerates Type 1 Diabetes in NOD/Ltj Mice

**DOI:** 10.3390/ijms27209182

**Published:** 2026-10-16

**Authors:** Yuandi Li, Keke He, Xunru Qu, Jie Gao, Rong Hu, Min Su

**Affiliations:** 1 Center for Tissue Engineering and Stem Cell Research, Translation Medicine Research Center, Guizhou Medical University, 6 Ankang Avenue, Guian New District, Guiyang 561113, China; lyd12230@163.com (Y.L.);; 2 Department of Human Histology, Embryology and Regenerative Medicine, Guizhou Medical University, 6 Ankang Avenue, Guian New District, Guiyang 561113, China; 3 Guizhou Biomanufacturing Laboratory, Guizhou Medical University, 6 Ankang Avenue, Guian New District, Guiyang 561113, China; 4 National Joint Local Engineering Laboratory for Cell Engineering and Biomedicine Technique, 6 Ankang Avenue, Guian New District, Guiyang 561113, China; 5 Key Laboratory of Adult Stem Cell Translational Research (Chinese Academy of Medical Sciences), Guizhou Medical University, 6 Ankang Avenue, Guian New District, Guiyang 561113, China; 6 Key Laboratory for Research on Autoimmune Diseases of Higher Education Schools in Guizhou Province, 6 Ankang Avenue, Guian New District, Guiyang 561113, China

**Keywords:** autoimmune regulators, NOD, Type 1 diabetes, embryonic stem cells, thymic epithelial cells

## Abstract

The autoimmune regulator (AIRE) is essential for central immune tolerance, but its role in thymic epithelial cell (TEC) differentiation and development remains incompletely understood. To address this, we knocked down AIRE in mouse embryonic stem cells (mESCs) and analyzed thymic epithelial progenitor (TEP) differentiation using RNA-sequencing and also performed thymic AIRE knockdown in NOD/Ltj mice to evaluate type 1 diabetes (T1D) pathogenesis. AIRE knockdown significantly reduced the proportion of K5^+^/K8^+^ TEPs among EpCAM1^+^ cells and altered the ECM–receptor interaction pathway, with downregulation of SPP1 and upregulation of Fn1 and CD44. Importantly, re-expression of AIRE using an shRNA-resistant rescue vector partially reversed these molecular changes and partially restored TEP differentiation. In vivo, thymic AIRE knockdown accelerated blood glucose elevation, aggravated insulitis, and reduced expression of T1D-associated TSAs (INS2, GAD67) and maturation markers (CD80, MHC-II). Importantly, the changes in SPP1, Fn1, and CD44 were consistent between in vitro and in vivo models. Furthermore, AIRE deficiency increased CD4^+^ single-positive thymocytes, reduced splenic Treg frequency, and enhanced antigen-specific T cell proliferation and activation. Collectively, these findings demonstrate that AIRE deficiency impairs TEP differentiation and alters the ECM-receptor pathway, while thymic AIRE deficiency compromises immune tolerance and accelerates T1D progression in NOD/Ltj mice.

## 1. Introduction

The autoimmune regulator (AIRE) gene was first identified in 1997 as the causative gene of autoimmune polyendocrinopathy-candidiasis-ectodermal dystrophy (APECED) [1,2]. Subsequent studies indicated that AIRE mRNA is highly expressed in the thymus [3,4], predominantly in medullary thymic epithelial cells (mTECs) [5]. AIRE plays a pivotal role in establishing central immune tolerance by mediating negative selection of thymocytes [6,7]. Mechanistically, AIRE drives the transcription of a broad repertoire of tissue-specific antigens (TSAs) in mTECs [8,9]. Loss or dysfunction of AIRE in mTECs results in diminished or absent TSA expression, which impairs negative selection, compromises the deletion of self-reactive T cells, and leads to aberrant T cell development [10,11]. AIRE is also recognized as a critical regulator of TEC maturation. mTEC development proceeds through three principal stages [12]: (1) MHClo, CD80lo, AIRE- immature mTECs; (2) MHChi, CD80hi, AIRE- pre-mature mTECs; and (3) MHChi, CD80hi, AIRE+ mature mTECs. However, the precise role of AIRE in TEC maturation and its underlying mechanisms remain incompletely elucidated.

In previous studies, we demonstrated that mouse embryonic stem cells (mESCs) can be efficiently induced to differentiate into thymic epithelial progenitor cells (TEPs) in vitro [13,14]. Upon transplantation, these mESC-derived TEPs can further differentiate into both cortical thymic epithelial cells (cTECs) and mTECs, reconstitute normal thymic architecture, and promote thymocyte development in recipients of syngeneic or allogeneic hematopoietic stem cell transplantation [15]. These findings provide a valuable cellular model and experimental platform for investigating the mechanisms by which AIRE regulates immune tolerance.

Type 1 diabetes (T1D), also termed insulin-dependent diabetes mellitus, is an organ-specific autoimmune disease characterized by T cell-mediated destruction of pancreatic β cells [16,17,18]. Non-obese diabetic (NOD) mice represent a well-established animal model of spontaneous T1D that closely recapitulates the immunological features, pathological progression, and clinical manifestations of human T1D [19,20].

In the present study, we employed an mESC-TEP differentiation model combined with AIRE shRNA lentiviral knockdown to investigate the role of AIRE in the in vitro differentiation of mESCs into TEPs. To strengthen the causal interpretation of the in vitro findings, we additionally performed an AIRE rescue experiment using an shRNA-resistant Aire expression vector in AIRE-knockdown mESCs. We also examined the effects of thymic AIRE knockdown on T cell development and T1D pathogenesis in NOD/Ltj mice. Our results show that AIRE knockdown reduces the differentiation efficiency of mESCs into TEPs and is associated with alterations in the ECM-receptor interaction pathway, whereas restoration of AIRE partially reverses these molecular changes and the impaired TEP differentiation phenotype. Furthermore, thymic AIRE deficiency compromises immune tolerance and accelerates T1D progression in NOD/Ltj mice.

## 2. Results

### 2.1. Establishment and Characterization of AIRE-Knockdown mESCs

**Figure 1:**
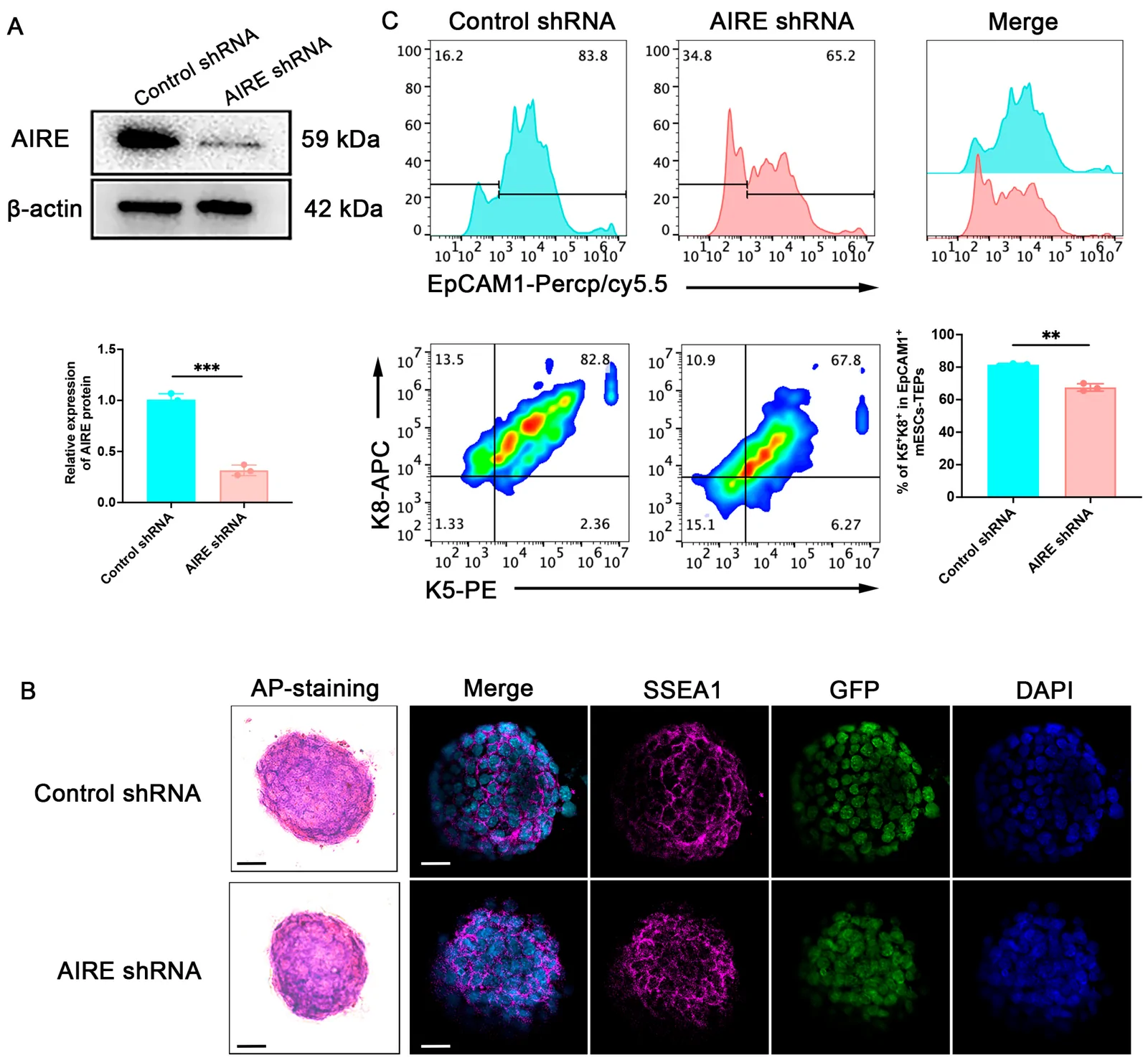
Characterization of AIRE shRNA mESCs. (**A**) AIRE protein expression in control shRNA and AIRE shRNA mESCs was determined by Western blot. Quantitative data are presented as individual biological replicates with mean ± SD (*n* = 3 biological replicates). Full-length blots are presented in Supplementary Figure S1. (**B**) Control shRNA and AIRE shRNA mESCs were evaluated for alkaline phosphatase (AP) activity, SSEA1 expression, and GFP reporter expression. GFP encoded by the lentiviral vector was used as a reporter of successful lentiviral transduction, whereas AP and SSEA1 were used to verify maintenance of the undifferentiated mESC state. Representative images are shown. Scale bar: 100 μm. (**C**) Control shRNA and AIRE shRNA mESCs were induced to differentiate into thymic epithelial progenitor cells (TEPs). EpCAM1^+^ cells were analyzed for K5 and K8 expression by flow cytometry. Quantitative data show the proportion of K5^+^/K8^+^ cells among EpCAM1^+^ cells and are presented as individual biological replicates with mean ± SD (*n* = 3 biological replicates), ** *p* < 0.01, and *** *p* < 0.001.

Undifferentiated ESCs can be genetically manipulated while maintaining their pluripotency and differentiation potential. To investigate the role of AIRE in TEP differentiation, we transduced mESCs with AIRE shRNA lentivirus and established stable monoclonal cell lines following puromycin selection. The control shRNA group was used as the primary comparator for the AIRE shRNA group throughout the in vitro analyses. Western blot analysis confirmed that AIRE protein expression was significantly reduced in the AIRE shRNA group compared with the control shRNA group (Figure 1A). Both AIRE shRNA and control shRNA mESCs exhibited positive alkaline phosphatase activity. Alkaline phosphatase staining was used as a commonly used marker of the undifferentiated state of mESCs, whereas GFP encoded by the lentiviral vector served as a reporter of successful lentiviral transduction. Immunofluorescence staining demonstrated that both groups expressed GFP and the pluripotency marker SSEA1 (Figure 1B), confirming that the mESCs maintained an undifferentiated state after genetic modification.

We next examined whether AIRE knockdown affects the differentiation of mESCs into TEPs in vitro. After induction, cells derived from both AIRE shRNA and control shRNA mESCs contained EpCAM1-positive cells that co-expressed K5 and K8, consistent with a TEP phenotype. However, flow cytometry analysis revealed that the proportion of K5^+^/K8^+^ double-positive cells among EpCAM1^+^ cells was significantly decreased in AIRE-knockdown mESC-TEPs relative to control shRNA mESC-TEPs (Figure 1C), supporting an inhibitory effect of AIRE deficiency on mESC-to-TEP differentiation.

### 2.2. AIRE Deficiency Alters the ECM-Receptor Interaction Pathway, and Restoration of AIRE Rescues Associated Molecular Changes and TEP Differentiation Defects

**Figure 2:**
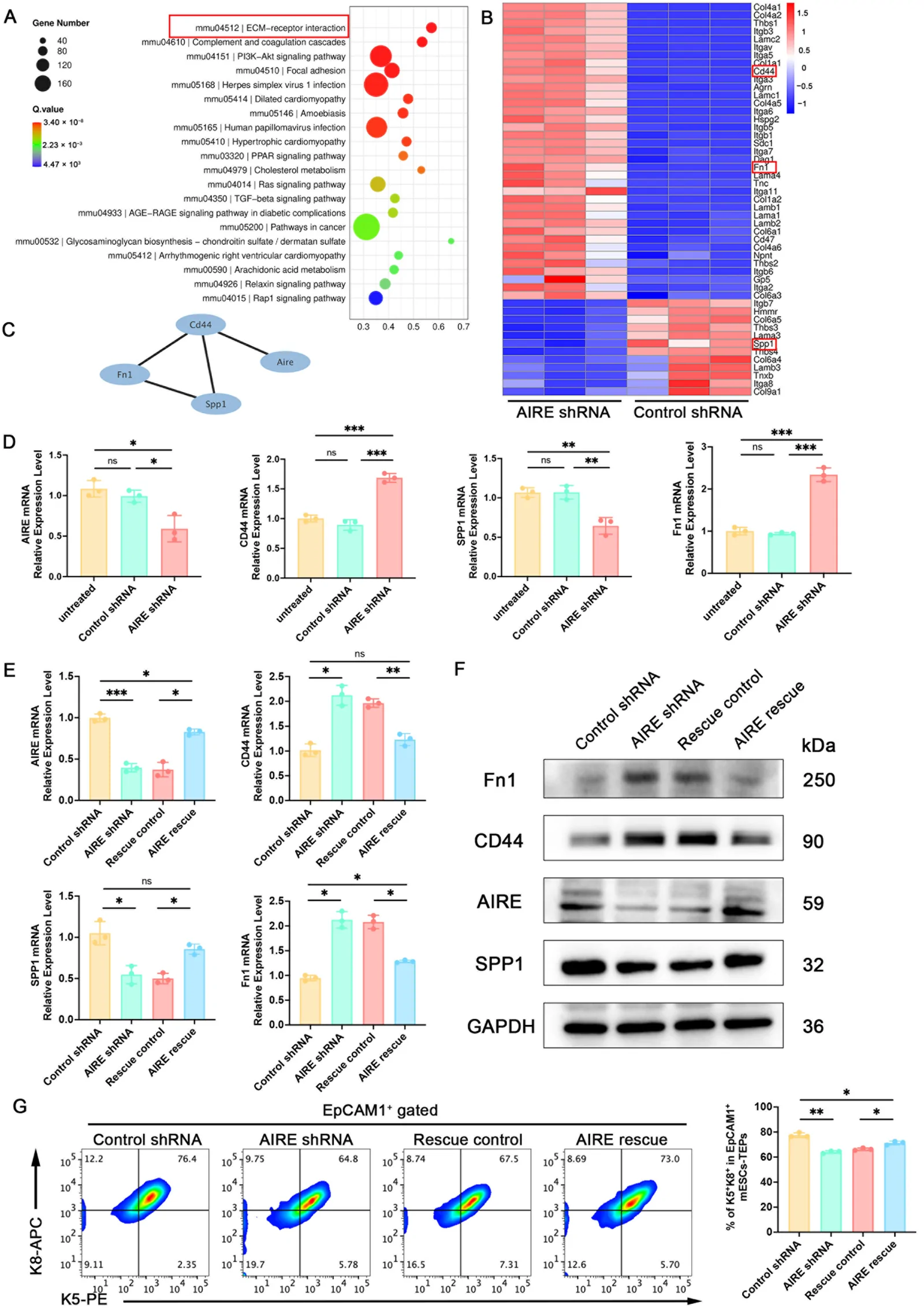
Transcriptomic analysis and rescue of ECM–receptor interaction-related alterations in AIRE-knockdown mESC-TEPs. (**A**) KEGG pathway enrichment analysis of differentially expressed genes between control shRNA and AIRE shRNA mESC-TEPs. (**B**) Heatmap of differentially expressed genes associated with the ECM–receptor interaction pathway. (**C**) Protein–protein interaction network involving AIRE, SPP1, FN1, and CD44. (**D**) qPCR analysis of Aire, Spp1, Fn1, and Cd44 expression in untreated, control shRNA, and AIRE shRNA mESC-TEPs. (**E**) qPCR analysis of Aire, Spp1, Fn1, and Cd44 expression in the control shRNA, AIRE shRNA, rescue control, and AIRE rescue groups. The rescue control group received the same vector backbone and transduction procedure as the AIRE rescue group but lacked functional Aire rescue expression. (**F**) Western blot analysis and densitometric quantification of AIRE, SPP1, FN1, and CD44 in the control shRNA, AIRE shRNA, rescue control, and AIRE rescue groups. (**G**) Flow cytometric analysis of K5 and K8 expression among EpCAM1^+^ cells following TEP differentiation in the control shRNA, AIRE shRNA, rescue control, and AIRE rescue groups. Restoration of AIRE expression increased the proportion of K5^+^/K8^+^ TEP-like cells compared with the rescue control group. Data are presented as mean ± SD (*n* = 3 biological replicates). ns, not significant; * *p* < 0.05, ** *p* < 0.01, and *** *p* < 0.001.

The differentiation of mESCs into TEPs is a complex and tightly regulated process. To investigate the molecular changes associated with AIRE deficiency during TEP differentiation, we performed RNA-seq analysis of differentiated mESC-TEPs from the control shRNA and AIRE shRNA groups. KEGG pathway enrichment analysis revealed that the ECM-receptor interaction pathway was among the most significantly altered pathways following AIRE knockdown; this pathway is known to play critical roles in cell adhesion, migration, differentiation, proliferation, and apoptosis (Figure 2A). Further analysis of differentially expressed genes within this pathway revealed that Spp1 and Aire were downregulated, whereas Fn1 and Cd44 were upregulated in AIRE-knockdown mESC-TEPs compared with control shRNA mESC-TEPs (Figure 2B,C).

To validate the transcriptomic findings, we examined the expression of Aire, Spp1, Fn1, and Cd44 by qPCR in the untreated, control shRNA, and AIRE shRNA groups. The control shRNA group was used as the primary comparator for the AIRE shRNA group, whereas the untreated group was included to evaluate potential effects associated with lentiviral transduction and selection. Consistent with the RNA-seq results, Aire and Spp1 expression was decreased, whereas Fn1 and Cd44 expression was increased in the AIRE shRNA group compared with the control shRNA group (Figure 2D). No significant differences were observed between the untreated and control shRNA groups for these genes.

To further determine whether these molecular alterations were attributable to AIRE deficiency, we performed a rescue experiment using an shRNA-resistant Aire expression vector in AIRE-knockdown mESCs, followed by the same mESC-to-TEP differentiation protocol. A matched rescue control vector with the same vector backbone but lacking functional Aire rescue expression was used as the rescue control. Restoration of AIRE expression was confirmed by qPCR and Western blotting. AIRE re-expression partially reversed the AIRE knockdown-associated changes in SPP1, FN1, and CD44 at both the mRNA and protein levels compared with the rescue control group (Figure 2E,F). We next examined whether AIRE restoration could rescue the impaired TEP differentiation phenotype. Flow cytometric analysis showed that the proportion of K5^+^/K8^+^ cells among EpCAM1^+^ cells, which was reduced following AIRE knockdown, increased following AIRE restoration compared with the rescue control group (Figure 2G). Together, these rescue data provide functional evidence that AIRE deficiency contributes to the ECM-associated molecular changes and impaired TEP differentiation observed in this model.

### 2.3. Thymic AIRE Knockdown Accelerates T1D Progression in NOD/Ltj Mice

**Figure 3:**
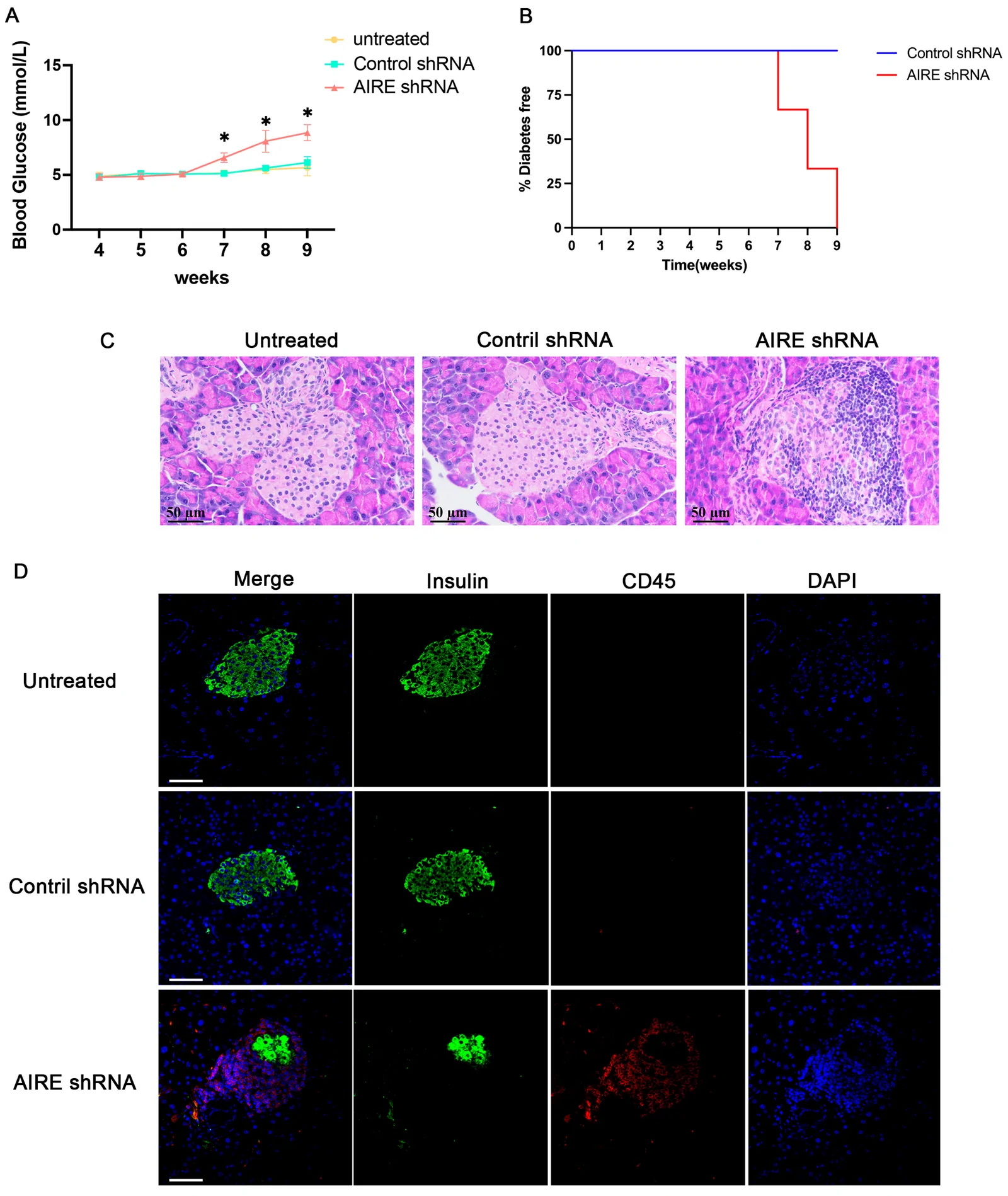
Thymic AIRE knockdown accelerates T1D progression in NOD/Ltj mice. NOD/Ltj mice received intrathymic injection of AIRE shRNA lentivirus (5 × 10^5^ TU/mL) or control shRNA lentivirus (5 × 10^5^ TU/mL), and T1D development was monitored for 28 days. (**A**) Blood glucose levels in NOD/Ltj mice over the 28-day observation period. (**B**) Diabetes-free rate in mice treated with Control shRNA or AIRE shRNA. (**C**) H&E staining of pancreatic sections from NOD/Ltj mice. Scale bars: 50 μm. Arrows indicate infiltrating lymphocytes. (**D**) Immunofluorescence staining for insulin (Ins) and CD45 in pancreatic sections from NOD/Ltj mice. Scale bars: 50 μm. * *p* < 0.05, Control shRNA versus AIRE shRNA.

To investigate the in vivo role of AIRE in thymic function and T1D pathogenesis, we injected AIRE shRNA lentivirus directly into the thymus of NOD/Ltj mice to knockdown thymic AIRE expression. Blood glucose levels were monitored weekly for four consecutive weeks. The results showed that mice in the AIRE shRNA group exhibited a more rapid elevation in blood glucose compared with the control and untreated groups, with some mice developing overt T1D as early as two weeks post-injection (Figure 3A). The diabetes-free rate remained at 100% in the Control shRNA group, while it decreased progressively in the AIRE shRNA group, reaching 0% by week 9 (Figure 3B).

Hematoxylin and eosin (H&E) staining of pancreatic sections revealed intact islet architecture with minimal lymphocyte infiltration in the untreated and control groups. In contrast, islets from the AIRE shRNA group exhibited disrupted structure with prominent lymphocytic infiltration, indicative of severe insulitis (Figure 3C). Immunofluorescence staining demonstrated that insulin-positive (Ins^+^) β cells constituted the majority of islet cells in the untreated and control groups, with negligible infiltration of CD45^+^ inflammatory cells. However, in the AIRE shRNA group, a substantial proportion of islets showed loss of Ins+ β cells accompanied by extensive CD45^+^ inflammatory cell infiltration (Figure 3D). These findings suggest that thymic AIRE knockdown exacerbates islet inflammation and accelerates T1D onset. Additionally, immunofluorescence staining confirmed reduced AIRE protein expression in the thymus of the AIRE shRNA group compared with the untreated and control groups.

### 2.4. Altered Expression of Thymic Maturation Markers, T1D-Associated TSAs, and ECM-Receptor Interaction Molecules Following Thymic AIRE Knockdown

**Figure 4:**
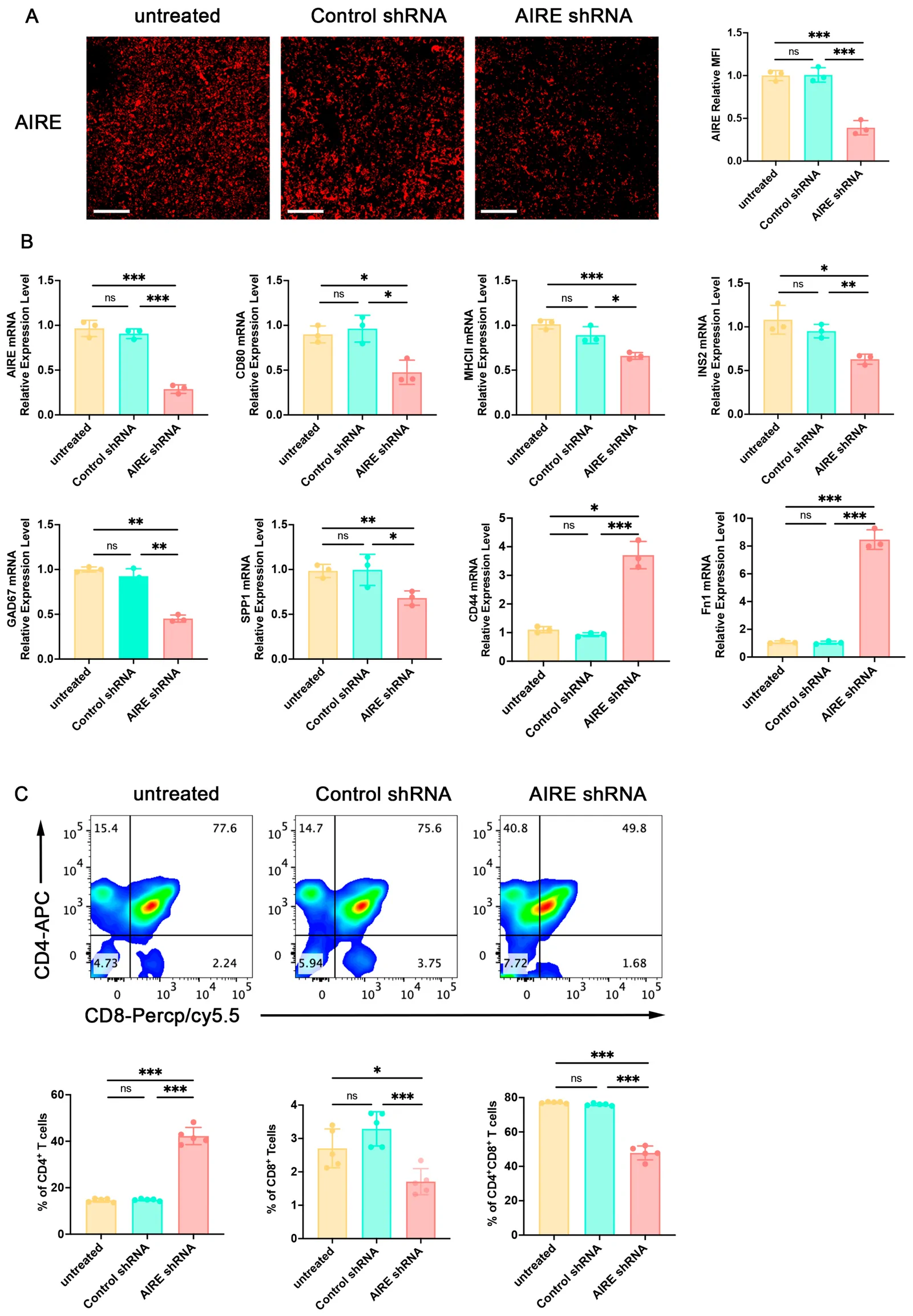
Effects of thymic AIRE knockdown on thymic gene expression and thymocyte subsets in NOD/Ltj mice. (**A**) Representative immunofluorescence staining of AIRE in thymic sections from untreated, control shRNA, and AIRE shRNA groups. Scale bars: 50 μm. (**B**) qPCR analysis of AIRE, CD80, MHCII, GAD67, INS2, SPP1, Fn1, and CD44 expression in thymic tissues from untreated, control shRNA, and AIRE shRNA groups. (**C**) Flow cytometric analysis of thymocyte subsets, including CD4^+^CD8^+^ double-positive (DP), CD4^+^ single-positive (CD4SP), and CD8^+^ single-positive (CD8SP) cells, in the three groups. Quantitative data are presented as mean ± SD (*n* = 5 mice per group). ns, not significant; * *p* < 0.05, ** *p* < 0.01, and *** *p* < 0.001.

Immunofluorescence analysis confirmed reduced AIRE protein expression in the thymic medulla of the AIRE shRNA group (Figure 4A). We further examined the mRNA expression levels of AIRE, mTEC maturation markers (CD80, MHC-II), and T1D-associated TSAs (GAD67, INS2) in the thymus by qPCR. The results showed that the expression of AIRE, CD80, MHC-II, GAD67, and INS2 was significantly decreased in the AIRE shRNA group compared with both untreated and control shRNA groups. Concurrently, Fn1 and CD44 expression was increased, while SPP1 expression was decreased in the thymus of AIRE shRNA-treated NOD/Ltj mice—remarkably consistent with the in vitro findings (Figure 4B).

### 2.5. Thymic AIRE Knockdown Increases CD4^+^ Single-Positive T Cells in NOD/Ltj Mice

We employed flow cytometry to assess the distribution of thymocyte subsets in the three groups. The results revealed a significant increase in the proportion of CD4^+^ single-positive (CD4SP) thymocytes and a concomitant decrease in CD4^+^CD8^+^ double-positive (DP) thymocytes in the AIRE shRNA group compared with the untreated and control groups (Figure 4C). These findings suggest that AIRE deficiency impairs negative selection, leading to defective clearance of CD4^+^ self-reactive T cells in the thymus.

### 2.6. Thymic AIRE Knockdown Promotes Peripheral T Cell Proliferation/Activation and Reduces Treg Frequency in NOD/Ltj

**Figure 5:**
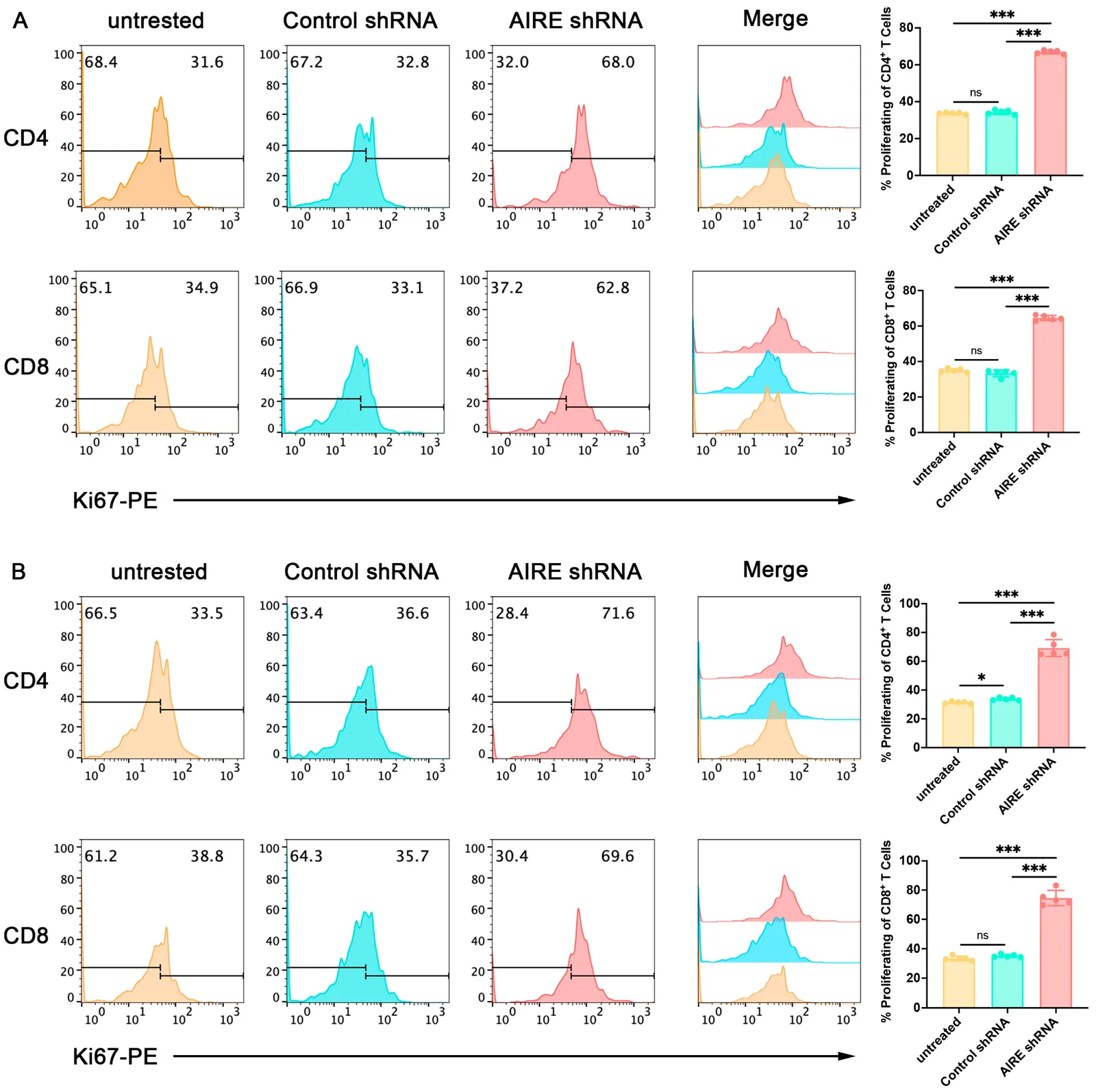
Effects of thymic AIRE knockdown on splenic T cell proliferation in NOD/Ltj mice. (**A**) Flow cytometric analysis of the percentage of Ki67^+^ cells among CD4^+^ and CD8^+^ T cells after stimulation with anti-CD3 for 5 days. (**B**) Flow cytometric analysis of the percentage of Ki67^+^ cells among CD4^+^ and CD8^+^ T cells after insulin stimulation. Data are presented as mean ± SD (*n* = 5 per group). ns, not significant; * *p* < 0.05 and *** *p* < 0.001.

To determine whether thymic AIRE knockdown affects peripheral T cell proliferation, we cultured splenocytes for 5 days under stimulation with anti-CD3 antibody or insulin antigen. Flow cytometry was performed to assess the proportion of Ki67^+^ proliferating cells among CD4^+^ and CD8^+^ T cells. The results showed that the AIRE shRNA group exhibited significantly enhanced proliferation of both CD4^+^ and CD8^+^ T cells, including insulin antigen-specific T cells, compared with the untreated and control groups (Figure 5A,B). These findings indicate that insulin-reactive CD4^+^ and CD8^+^ T cells are present in the peripheral lymphoid organs of NOD/Ltj mice and that thymic AIRE deficiency promotes the expansion of these self-reactive T cells.

**Figure 6:**
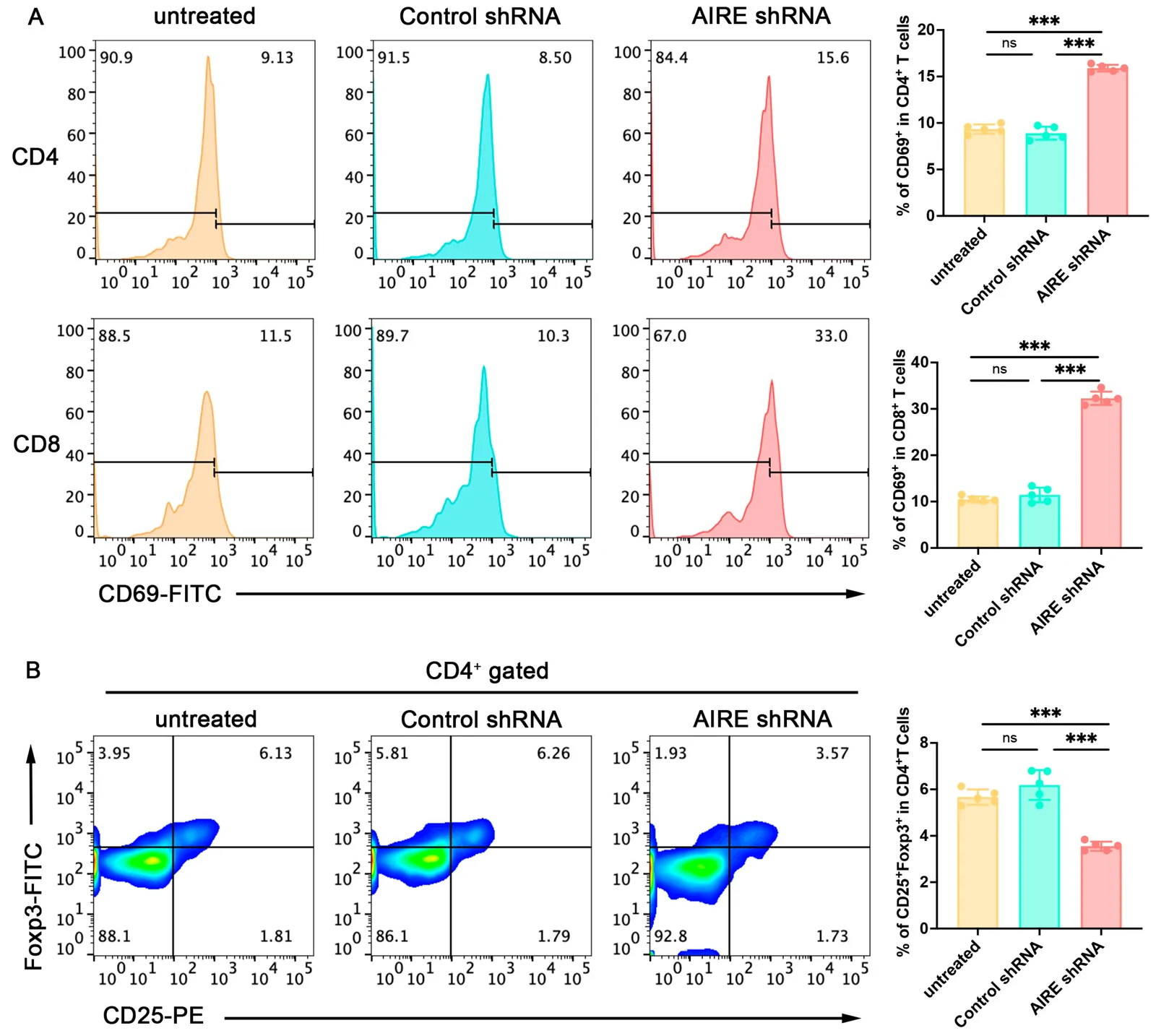
Effects of thymic AIRE knockdown on splenic T cell activation and Treg frequency in NOD/Ltj mice. (**A**) Flow cytometric analysis of CD69 expression in splenic CD4^+^ and CD8^+^ T cells. (**B**) Flow cytometric analysis of CD4^+^CD25^+^Foxp3^+^ regulatory T cells (Tregs) in the spleen. Quantitative data are presented as mean ± SD (*n* = 5 mice per group). ns, not significant, *** *p* < 0.001.

CD69 is a well-recognized early activation marker of T cells. Flow cytometry analysis demonstrated that CD69 expression was significantly upregulated on both CD4+ and CD8^+^ T cells from the AIRE shRNA group (Figure 6A), indicating that thymic AIRE deficiency leads to enhanced activation of peripheral T cells.

Regulatory T cells (Tregs), characterized as CD4^+^CD25^+^Foxp3^+^ cells, play a critical role in maintaining peripheral immune tolerance. We measured the frequency of Tregs in the spleen of mice from each group. The percentage of splenic CD4^+^CD25^+^Foxp3^+^ Tregs was significantly decreased in the AIRE shRNA group compared with the untreated and control shRNA groups (Figure 6B). These data demonstrate a reduced splenic Treg frequency following thymic AIRE knockdown.

## 3. Discussion

In the present study, we demonstrate that AIRE knockdown in mESCs reduces the efficiency of TEP differentiation and alters the ECM-receptor interaction pathway. Importantly, restoration of AIRE expression using an shRNA-resistant Aire rescue vector partially reversed the associated molecular changes and improved the impaired TEP differentiation phenotype, providing functional support for the involvement of AIRE in these processes. In vivo, thymic AIRE deficiency in NOD/Ltj mice impairs thymic negative selection, promotes peripheral T cell proliferation and activation, and reduces splenic Treg frequency, collectively accelerating T1D pathogenesis. These findings provide novel functional insights into the role of AIRE in both TEC development and immune tolerance.

The thymus serves as the primary organ for T cell development and maturation [21], where thymocytes undergo both positive and negative selection. Positive selection ensures MHC restriction, while negative selection eliminates self-reactive T cell clones to establish central tolerance [22]. mTECs constitute a critical component of the thymic microenvironment and present TSAs on MHC molecules to developing thymocytes. T cell clones with high affinity for TSA-MHC complexes are deleted via negative selection, whereas those with low affinity escape to the periphery and may become self-reactive T cells, potentially triggering autoimmune responses [8,23]. AIRE is highly expressed in mTECs and drives the transcription of a broad array of TSA-encoding genes [9].

In this study, we employed NOD/Ltj mice, a well-established model of spontaneous T1D, to investigate the in vivo consequences of thymic AIRE deficiency. We found that AIRE-knockdown NOD/Ltj mice exhibited severe disruption of islet architecture, with extensive CD45^+^ inflammatory cell infiltration into islets and concomitant loss of Ins^+^ β cells. In contrast, pancreatic islets from untreated and control shRNA mice maintained normal structure with minimal inflammatory infiltration. At the molecular level, thymic AIRE knockdown resulted in reduced expression of CD80 and MHC-II, as well as decreased levels of T1D-associated TSAs, including INS2 and GAD67. The increased proportion of CD4SP thymocytes and decreased DP thymocytes, together with enhanced peripheral T cell proliferation and activation, collectively indicate that AIRE deficiency compromises TSA expression and perturbs T cell development, leading to impaired thymic negative selection and defective peripheral tolerance. These findings are consistent with the established paradigm that AIRE deficiency in mTECs leads to reduced or absent TSA expression, impaired negative selection, and defective deletion of self-reactive T cells. Additionally, we observed a significant reduction in splenic Treg frequency in AIRE-knockdown mice, further suggesting that peripheral immune tolerance is compromised.

TECs originate from definitive endoderm (DE) and comprise two major subsets—cTECs and mTECs—both of which arise from common thymic epithelial progenitor cells [24]. Embryonic stem cells possess unlimited self-renewal capacity and pluripotency, capable of differentiating into cell types representing all three germ layers [25]. Our group has previously demonstrated that mESCs can be efficiently induced to differentiate into TEPs in vitro. When transplanted into recipients of syngeneic or allogeneic hematopoietic stem cell transplantation, mESC-derived TEPs can further differentiate into both cTECs and mTECs, reconstitute normal thymic architecture, and support thymocyte development.

To determine the effect of AIRE on the in vitro differentiation of mESCs into TEPs, we transduced mESCs with AIRE shRNA lentiviral vectors and induced TEP differentiation. Our results show that AIRE knockdown significantly reduces TEP differentiation efficiency. Transcriptomic analysis identified the ECM-receptor interaction pathway as one of the major pathways altered following AIRE knockdown. Specifically, AIRE knockdown in mESCs led to downregulation of Spp1 and upregulation of CD44 and Fn1 within the ECM-receptor interaction pathway. Notably, re-expression of AIRE using an shRNA-resistant Aire rescue vector partially reversed these changes at both the mRNA and protein levels and increased the proportion of K5^+^/K8^+^ TEP-like cells compared with the rescue control group. These rescue findings strengthen the functional association between AIRE deficiency, ECM-related molecular alterations, and impaired TEP differentiation. Importantly, similar changes in Cd44, Spp1, and Fn1 expression were observed in the thymus of AIRE-knockdown mice, supporting the biological relevance of these findings in both the in vitro and in vivo models.

CD44 is a widely distributed cell surface glycoprotein that mediates cell-extracellular matrix adhesion, lymphocyte homing, T cell activation, and cell migration [26,27,28]. SPP1 (Osteopontin) regulates leukocyte adhesion and migration primarily through β1 and β3 integrin receptors and CD44 receptors [29,30]. Previous studies have shown that SPP1 interaction with integrin and CD44 receptors on T cells promotes Th1 differentiation and inhibits Th2 development, thereby enhancing cellular immunity [31,32]. However, the precise molecular mechanisms linking AIRE to SPP1, FN1, and CD44 expression during TEC differentiation remain to be further elucidated. Several limitations should also be considered. The identification of mESC-derived TEPs in this study was based mainly on EpCAM1 together with K5/K8 expression, and additional phenotypic or functional markers could further strengthen the characterization of the differentiated cells. In addition, although all treated mice underwent the same intrathymic lentiviral delivery procedure, transduction efficiency was not quantitatively assessed in each individual animal. Despite these limitations, our findings provide functional evidence supporting a role for AIRE in mESC-to-TEP differentiation and its association with thymic immune tolerance and T1D progression.

## 4. Materials and Methods

### 4.1. Mice

Female 4-week-old NOD/Ltj mice were obtained from Beijing HFK Bioscience Co., Ltd. (Beijing, China). Animals were maintained under specific pathogen-free conditions with controlled temperature and light-dark cycles and had free access to food and water. All animal procedures were approved by the Ethics Committee of Guizhou Medical University (Approval No. 2000030; Approval Date: 26 March 2020) and were conducted in accordance with institutional and national guidelines.

### 4.2. Cell Culture and Differentiation

B6 mESCs were cultured in the ESGRO Complete PLUS Clonal Grade Medium (Sigma-Aldrich, St. Louis, MO, USA). To differentiate mESCs into definitive endoderm (DE), the cells were cultured in DMEM medium supplemented with 15% FBS. The combination of activin A (50 ng/mL) and Noggin (200 ng/mL) was added to the cultures for 2 days. For the differentiation of mESCs-DE into TEPs, the cells were cultured in the presence of human FGF7 (20 ng/mL), FGF10 (20 ng/mL), EGF (50 ng/mL), and BMP-4 (20 ng/mL) for another 10 days. All experimental groups underwent the same differentiation protocol.

### 4.3. AIRE Knockdown and Rescue in mESCs

B6 mESCs were transduced with AIRE shRNA lentivirus (2 × 10^7^ TU/mL; Chongqing Biomedicine Biotechnology Co., Ltd., Chongqing, China) or a corresponding non-targeting control shRNA lentivirus supplied by the same manufacturer. The Aire shRNA target sequence used in the functional experiments was 5′-CAGCAGGTGTTTGAGTCAGGAAGAT-3′. Following transduction, cells were selected with puromycin (0.5 µg/mL; Solarbio, Beijing, China) for 7 days. For rescue experiments, AIRE-knockdown mESCs were transduced with an shRNA-resistant mouse Aire expression vector in which synonymous nucleotide substitutions were introduced within the Aire shRNA recognition region while preserving the encoded AIRE amino-acid sequence. A matched rescue control vector with the same vector backbone and transduction procedure but lacking functional Aire rescue expression was used as the rescue control. AIRE restoration was verified by qPCR and Western blotting, after which rescued cells were subjected to the same mESC-to-TEP differentiation protocol described above.

### 4.4. RNA-Seq Sequencing and Data Analysis

Differentiated mESC-TEPs from the control shRNA and AIRE shRNA groups were collected for RNA-seq analysis, with three biological replicates per group, each derived from an independent differentiation experiment. Total RNA was extracted using TRIzol reagent (Thermo Fisher Scientific, Waltham, MA, USA, Cat. No. 15596018). Strand-specific RNA libraries were constructed and sequenced on an Illumina NovaSeq 6000 platform (LC Biotechnology Co., Ltd., Hangzhou, China) using 150 bp paired-end sequencing. Approximately 41.34–46.94 million raw reads per sample were generated, of which approximately 40.19–45.44 million clean reads per sample were retained after quality filtering.

Raw reads were filtered using Cutadapt (v1.9) to remove adapter-containing reads, poly-A/poly-G-containing reads, reads with >5% unknown bases, and reads with >20% low-quality bases (Q ≤ 20), and were aligned to the mouse reference genome using HISAT2 (v2.2.1). Transcript assembly and expression quantification were performed using StringTie (v2.1.6). Differential expression analysis was conducted using DESeq2 based on raw read counts with the default median-of-ratios normalization and a design formula of ~ condition, with the control shRNA group as the reference. Independent filtering and Cook’s distance cutoff were disabled. *p* values were adjusted using the Benjamini–Hochberg method, and genes with |log2 fold change| ≥ 1 and q < 0.05 were considered differentially expressed. GO and KEGG enrichment analyses were performed using the Gene Ontology and KEGG databases (version/date: May 2021). Protein–protein interaction analysis was performed using the STRING database (accessed in May 2021). The RNA-seq data generated in this study have been deposited in the NCBI Gene Expression Omnibus (GEO) under accession number GSE350412.

### 4.5. In Vivo T1D Model

For intrathymic AIRE knockdown, mice were assigned to untreated, control shRNA, and AIRE shRNA groups (n = 5 per group). Mice in the control shRNA and AIRE shRNA groups underwent surgical exposure of the thymus and received intrathymic injection of the corresponding lentiviral vector. Specifically, 5 µL of control shRNA lentivirus or AIRE shRNA lentivirus was injected into the anterior-superior region of each thymic lobe using a 0.1 mL syringe fitted with a 28-gauge needle. Mice in the untreated group received no intrathymic lentiviral injection. Random blood glucose was measured using an Abbott glucose meter, and T1D onset was defined as blood glucose ≥11.1 mmol/L on two consecutive measurements. On day 28, mice were anesthetized by intraperitoneal injection of pentobarbital sodium (40 mg/kg) and euthanized by excess carbon dioxide.

### 4.6. Flow Cytometry Analysis

Single-cell suspensions were prepared and stained with the indicated fluorochrome-conjugated antibodies. For intracellular antigens, cells were fixed in 4% paraformaldehyde for 10 min and permeabilized with 0.2% Triton X-100 for 20 min at 4 °C before staining. Antibodies used for immune-cell phenotyping included CD4 (BioLegend, San Diego, CA, USA, Cat. No. 100516), CD8 (BioLegend, Cat. No. 100734), CD25 (BioLegend, Cat. No. 101904), Foxp3 (BioLegend, Cat. No. 126406), CD69 (BioLegend, Cat. No. 310903), and Ki67 (BioLegend, Cat. No.350504). TEP differentiation was assessed using EpCAM1 (BioLegend, Cat. No. 118220), keratin 5 (K5; GeneTex, Irvine, CA, USA, Cat. No. GTX60580), and keratin 8 (K8; GeneTex, Cat. No. GTX112975). In the rescue experiment, control shRNA, AIRE shRNA, rescue control, and AIRE rescue mESC-derived cells were analyzed after TEP differentiation, and EpCAM1^+^ cells were evaluated for K5/K8 co-expression. The percentage of EpCAM1^+^K5^+^K8^+^ cells was used as the TEP differentiation readout. APC- or PE-conjugated anti-mouse/anti-rabbit IgG secondary antibodies were obtained from Abcam (Cambridge, UK, Cat. Nos. ab72465, ab130786, respectively). Samples were acquired on a FACSCalibur flow cytometer (BD Biosciences, Franklin Lakes, NJ, USA) and analyzed with FlowJo software v10.8.1 (FlowJo, LLC, Ashland, OR, USA). Flow cytometric analysis was performed as previously described [33], with minor modifications.

### 4.7. Reverse Transcription Polymerase Chain Reaction and Quantitative Real-Time Reverse Transcription Polymerase Chain Reaction

Total RNA was isolated from cultured cells or thymic tissue using TRIzol reagent (Thermo Fisher Scientific, Cat. No. 15596018) according to the manufacturer’s instructions. cDNA was synthesized using MonScript™ RTIII All-in-One Mix with dsDNase (Monad, Wuhan, China, Cat. No. MR05101M). Equal amounts of cDNA were subjected to quantitative real-time PCR using MonAmp™ SYBR^®^ Green qPCR Mix (Monad, Cat. No. MQ10101S) on a 7500 Real-Time PCR System (Applied Biosystems, Waltham, MA, USA). Cycling conditions were 95 °C for 30 s, followed by 40 cycles of 95 °C for 10 s and 60 °C for 35 s. Primer sequences are provided in Supplementary Table S1.

### 4.8. Western Blot

Total cellular protein was separated on 8% or 10% SDS-polyacrylamide gels, as appropriate, and transferred to polyvinylidene fluoride membranes. Membranes were blocked and incubated with primary antibodies against AIRE (R&D Systems, Minneapolis, MN, USA, Cat. No. MAB6184), SPP1 (Proteintech, Wuhan, China, Cat. No. 30200-1-AP), FN1 (Proteintech, Cat. No. 15613-1-AP), and CD44 (Proteintech, Cat. No. 30854-1-AP), followed by appropriate HRP-conjugated secondary antibodies (NEOBIOSCIENCE, Shenzhen, China, Cat. No. ANR03-1, abmart, Shanghai, China, Cat. No. M21002).

### 4.9. Histopathology

Thymus and pancreas tissues were collected, fixed in 10% formaldehyde for 24 h, embedded in paraffin, and sectioned. Pancreatic sections were stained with hematoxylin and eosin for histological evaluation. For immunofluorescence, thymic sections were stained with rabbit anti-mouse K5 polyclonal antibody (GeneTex, Cat. No. GTX60580), mouse anti-mouse K8 monoclonal antibody (GeneTex, Cat. No. GTX112975), and rat anti-AIRE monoclonal antibody (R&D Systems, Cat. No. MAB6184). Pancreatic sections were stained with rabbit anti-mouse insulin monoclonal antibody (Abcam, Cat. No. ab181547) and rat anti-mouse CD45 monoclonal antibody (Novus, Centennial, CO, USA, Cat. No. NB100-77417). mESCs were stained with mouse anti-mouse SSEA1 monoclonal antibody (GeneTex, Cat. No. GTX48035). Alexa Fluor 488- (Abcam, Cat. No. ab150113), 594- (Abcam, Cat. No. ab150160), or 647-conjugated secondary antibodies (Abcam, Cat. No. ab150123) were used as appropriate. Fluorescence images were acquired using an Olympus confocal microscope, and scale bars were included in all representative immunofluorescence micrographs.

### 4.10. Statistical Analysis

Statistical analyses were performed using SPSS version 21.0. (IBM Corp., Armonk, NY, USA) Data are presented as mean ± standard deviation (SD), and individual biological replicates are shown in quantitative graphs. Comparisons between two groups were performed using two-sided Student’s *t*-tests, while comparisons among multiple groups were performed using one-way analysis of variance (ANOVA), as appropriate. A *p* value < 0.05 was considered statistically significant. ns, not significant; * *p* < 0.05, ** *p* < 0.01, and *** *p* < 0.001.

## Data Availability

The RNA-seq data generated in this study have been deposited in the NCBI Gene Expression Omnibus (GEO) under accession number GSE350412.

## Supplementary Materials

The following supporting information can be downloaded at: https://www.mdpi.com/article/10.3390/ijms27209182/s1.

## Abbreviations

The following abbreviations are used in this manuscript:

AIRE	Autoimmune regulator
AP	Alkaline phosphatase
APECED	Autoimmune polyendocrinopathy-candidiasis-ectodermal dystrophy
BMP-4	Bone morphogenetic protein 4
cTEC	Cortical thymic epithelial cell
DE	Definitive endoderm
DP	Double-positive
ECM	Extracellular matrix
EGF	Epidermal growth factor
FBS	Fetal bovine serum
FGF7	Fibroblast growth factor 7
FGF10	Fibroblast growth factor 10
GFP	Green fluorescent protein
GO	Gene Ontology
H&E	Hematoxylin and eosin
HRP	Horseradish peroxidase
IF	Immunofluorescence
KEGG	Kyoto Encyclopedia of Genes and Genomes
mESC	Mouse embryonic stem cell
MHC-II	Major histocompatibility complex class II
mTEC	Medullary thymic epithelial cell
NOD	Non-obese diabetic
qPCR	Quantitative real-time polymerase chain reaction
RNA-seq	RNA sequencing
SD	Standard deviation
shRNA	Short hairpin RNA
SP	Single-positive
SSEA1	Stage-specific embryonic antigen 1
T1D	Type 1 diabetes
TEC	Thymic epithelial cell
TEP	Thymic epithelial progenitor
Treg	Regulatory T cell
TSA	Tissue-specific antigen
