# Supplementary material for: AIRE Deficiency Impairs Thymic Epithelial Progenitor Differentiation from Mouse Embryonic Stem Cells and Accelerates Type 1 Diabetes in NOD/Ltj Mice
Source: Int J Mol Sci. 2026 Oct 16;27(20):9182. doi: 10.3390/ijms27209182 (PMC13705361; doi:10.3390/ijms27209182)
Supplement: Supplementary file 1 [file ijms-27-09182-s001.zip › ijms-4571066-supplementary.pdf]

Supplementary Figure

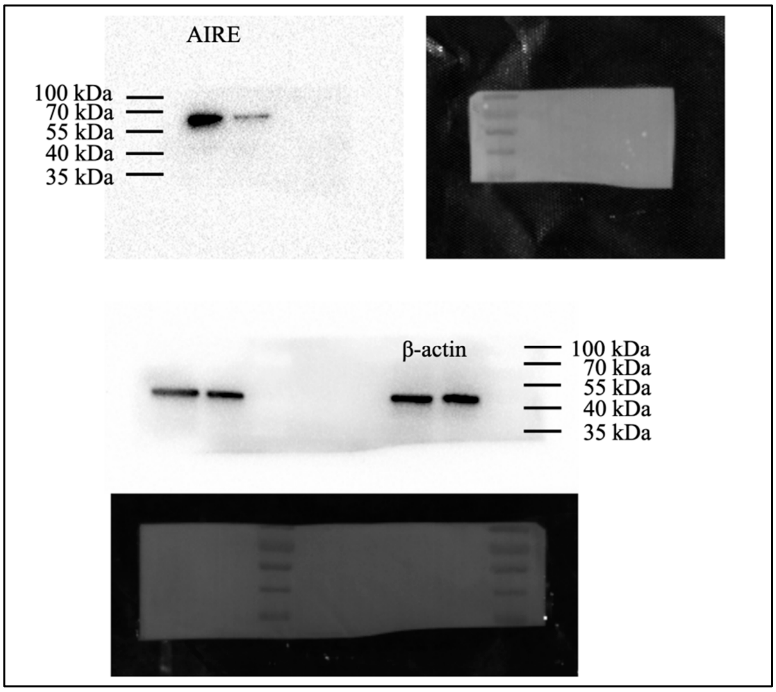

Figure S1. Uncropped full-length blol.

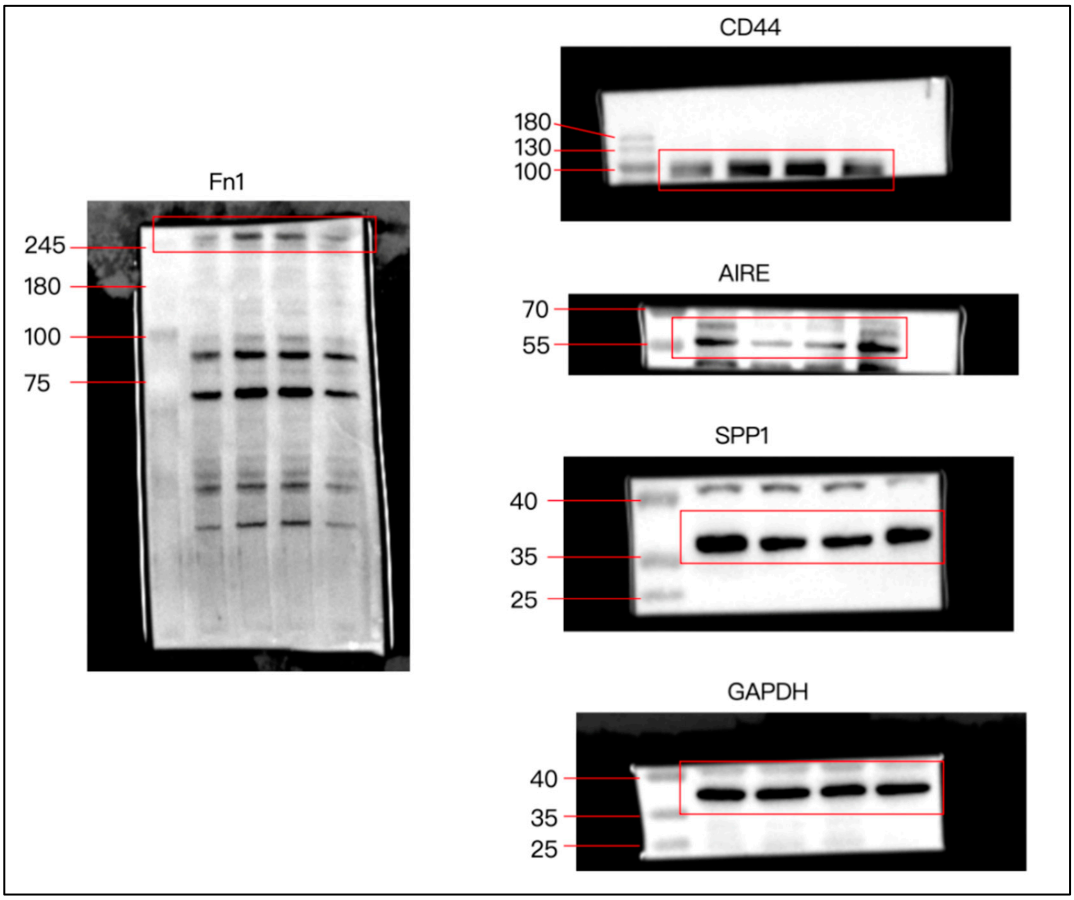

Figure S2. Uncropped full-length blol.

Supplementary Table

Table S1. Primer sequences used for quantitative real-time PCR (qPCR)

Gene	Forward primer (5′–3′)	Reverse primer (5′–3′)
Aire	AGGTCAGCTTCAGAGAAAACCA	TCATTCCCAGCACTCAGTAGA
Ins2	ACCTTCAGACCTTGGCACTG	GCTGGGTAGTGGTGGGTCTA
Cd80	CAAAGAAAAACCAGGAGGGCG	TTTGAAGTCTCGTGGTGAGC
MhcII	ACCAACGGGACGCAGCGCAT	CAAGCCGCCGCAGGGAGGTG
Gad67	CTCAGGCTGTATGTCAGATGTTC	AAGCGAGTCACAGAGATTGGTC
Cd44	ATCCTCGTCACGTCCAACAC	AGCGAGTACCATCACGGTTG
Spp1	TCCCTCGATGTCATCCCTGT	ATCACATCCGACTGATCGGC
Fn1	TTGGATTCTGCCCAATGGCT	ACACACGTGCACCTCATCAT
Gapdh	TGTTTCCTCGTCCCGTAG	TGTTTCCTCGTCCCGTAG
